# Synthesis, crystal structure and thermal properties of di­aqua­bis­(4-methyl­pyridine-κ*N*)bis­(thio­cyanato-κ*N*)cobalt(II)

**DOI:** 10.1107/S2056989025003469

**Published:** 2025-04-24

**Authors:** Christian Näther, Jan Boeckmann

**Affiliations:** a Institut für Anorganische Chemie, Universität Kiel, Germany; University of Aberdeen, United Kingdom

**Keywords:** synthesis, crystal structure, cobalt thio­cyanate, 4-methyl­pyridine, thermal properties

## Abstract

The reaction of Co(NCS)_2_ and 4-methyl­pyridine (C_6_H_7_N) in water leads to the formation of light-blue single crystals of the title compound, [Co(NCS)_2_(C_6_H_7_N)_2_(H_2_O)_2_]. The asymmetric unit consists of one Co^II^ cation (site symmetry 1) as well as one thio­cyanate anion, one 4-methyl­pyridine coligand and one water mol­ecule in general positions to generate *trans*-CoN_4_O_2_ octa­hedra. In the crystal, the complexes are linked by O—H⋯S hydrogen bonds into a layered network. Powder X-ray diffraction (PXRD) shows that a pure sample has been obtained. Upon heating, the title compound loses its water mol­ecules and transforms into Co(NCS)_2_(C_6_H_7_N)_2_, which is already reported in the literature.

## Abstract 2

In the title compound, the Co^II^ cations are octa­hedrally coordinated by two N-bonded thio­cyanate anions, two 4-methyl­pyridine ligands and two water mol­ecules into discrete complexes that are linked by O—H⋯S hydrogen bonds into layers. Upon heating, the title compound loses the water mol­ecules and transforms into Co(NCS)_2_(C_6_H_7_N)_2_, which is already reported in the literature

## 1. Chemical context

The synthesis of new coordination compounds is still an important field in inorganic chemistry. In most cases, they are prepared in solution but there are synthetic alternatives such as mol­ecular milling (Braga *et al.*, 2005 ▸, 2006 ▸; James *et al.*, 2012 ▸; Do & Friščić, 2017 ▸; Stolar *et al.*, 2017 ▸) or reactions in melts (Müller-Buschbaum, 2005 ▸; Höller & Müller-Buschbaum, 2008 ▸; Zurawski *et al.*, 2012 ▸). We have also developed a new route, which is based on thermal ligand removal, that in the beginning was used for the preparation of new transition-metal halide compounds (Näther *et al.*, 2001 ▸, 2002 ▸). Later, this method was used for the synthesis of transition-metal thio- and seleno­cyanate coordination compounds, which were of inter­est not only because of their versatile structural behavior but also because of their promising magnetic properties. Following this route, discrete complexes with the composition *M*(NCS)_2_(*L*)_4_ (*M* = Mn, Fe, Co, Ni and *L* = neutral N-donor coligand) are heated in a thermobalance, which mostly leads to a stepwise removal of the coligands and the transformation into new compounds with the composition *M*(NCS)_2_(*L*)_2_, in which the metal cations are linked into chains or layers (Werner *et al.*, 2015 ▸; Neumann *et al.*, 2018 ▸; Jochim *et al.*, 2020*a* ▸). In this context, Co(NCS)_2_ compounds with chain structures are of special inter­est, because they can show one-dimensional or three-dimensional ferromagnetic ordering (Mautner *et al.*, 2018 ▸; Rams *et al.*, 2017 ▸, 2020 ▸; Jochim *et al.*, 2020*b* ▸).

However, in some cases the thermogravimetric curves are not well resolved and the isolation of a pure inter­mediate phase is difficult or even impossible to achieve. In such cases, the usage of precursors, consisting of simple solvato complexes with the composition *M*(NCS)_2_(*L*1)_2_(*L*2)_2_ (*L*1 = monocoordinating neutral N-donor coligand, *L*2 = *e.g.*, H_2_O, MeOH, EtOH, MeCN) is of advantage. If they are heated, the solvent is lost in the beginning in a well-resolved step and the desired inter­mediate phase is obtained pure (Näther *et al.*, 2013 ▸). In this context, we have reported on two isomers of Co(NCS)_2_(4-chloro­pyridine)_2_ in which the metal cations are octa­hedrally coordinated and linked by pairs of thio­cyanate anions into chains (Böhme *et al.*, 2020 ▸). In the triclinic isomer, an all-*trans* configuration is observed, leading to the formation of linear chains, whereas in the monoclinic isomer an alternating all-*trans* and *cis*–*cis*–*trans* configurations are observed, which results in the formation of corrugated chains. The monoclinic form is obtained from solution, whereas the triclinic form can be obtained by thermal decomposition of the precursor complex Co(NCS)_2_(4-chloro­pyridine)_2_(H_2_O)_2_. Solvent-mediated conversion experiments reveal that the monoclinic form with corrugated chains is thermodynamically stable at room-temperature.
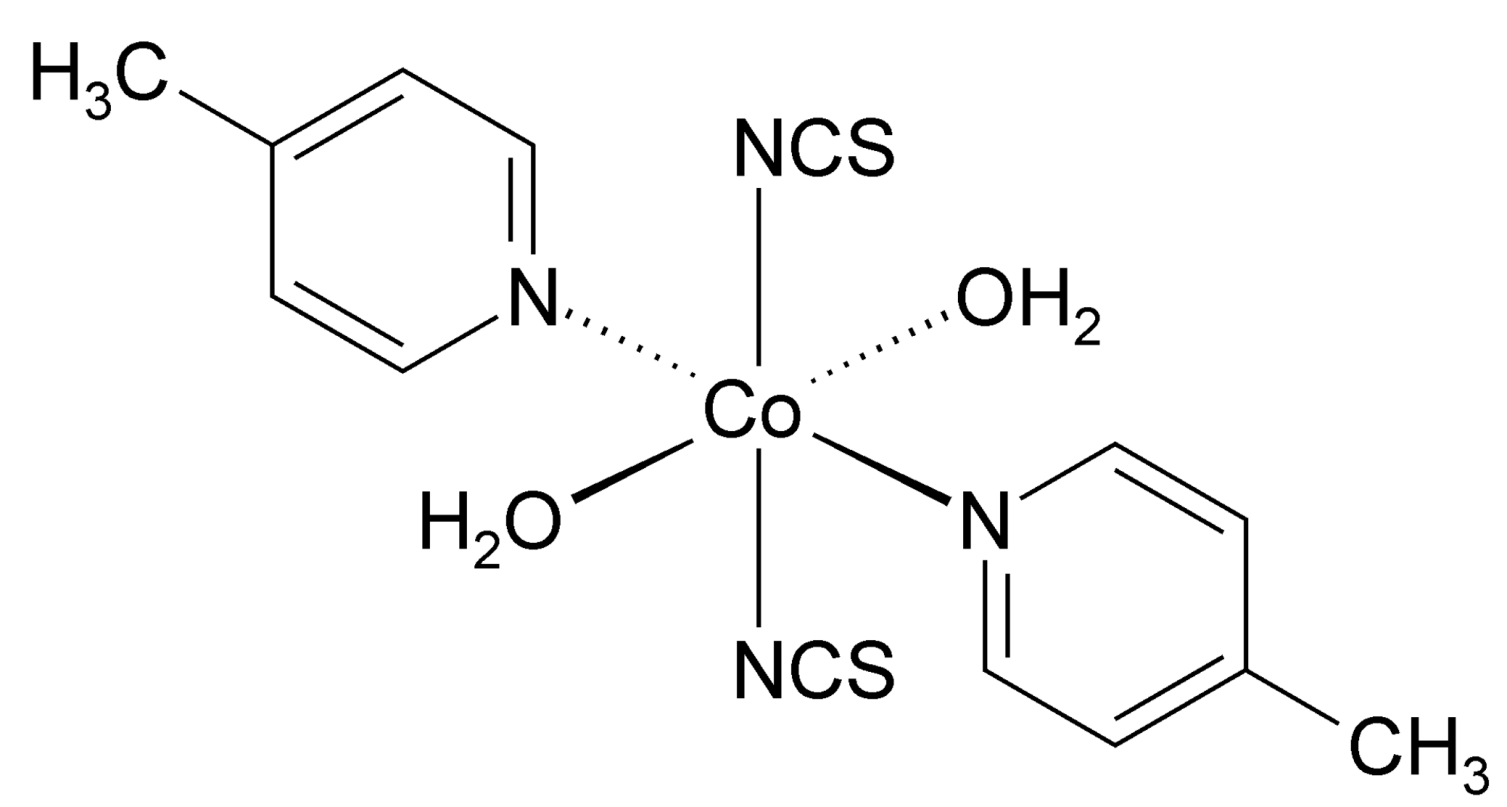


In a continuation of this work, we reported the synthesis and crystal structure of the analogous compound Co(NCS)_2_(4-methyl­pyridine)_2_ in which the chlorine atom is replaced by a methyl group (Näther & Boeckmann, 2025 ▸). Based on the chloro–methyl exchange rule (Desiraju & Sarma, 1986 ▸), we expected the formation of a similar crystal structure that might be isotypic to one of the two forms of Co(NCS)_2_(4-chloro­pyridine)_2_. A single-crystal structure determination of the 4-methyl­pyridine compound proved that it is isotypic to the monoclinic form of Co(NCS)_2_(4-chloro­pyridine)_2_ that consists of corrugated chains. Based on the results obtained for the 4-chloro­pyridine ligands, it was of inter­est if an isomer with linear chains can be prepared by thermal decomposition of a suitable precursor with the composition Co(NCS)_2_(4-chloro­pyridine)_2_(H_2_O)_2_, which is not reported in the literature.

## 2. Structural commentary

**Figure 1:**
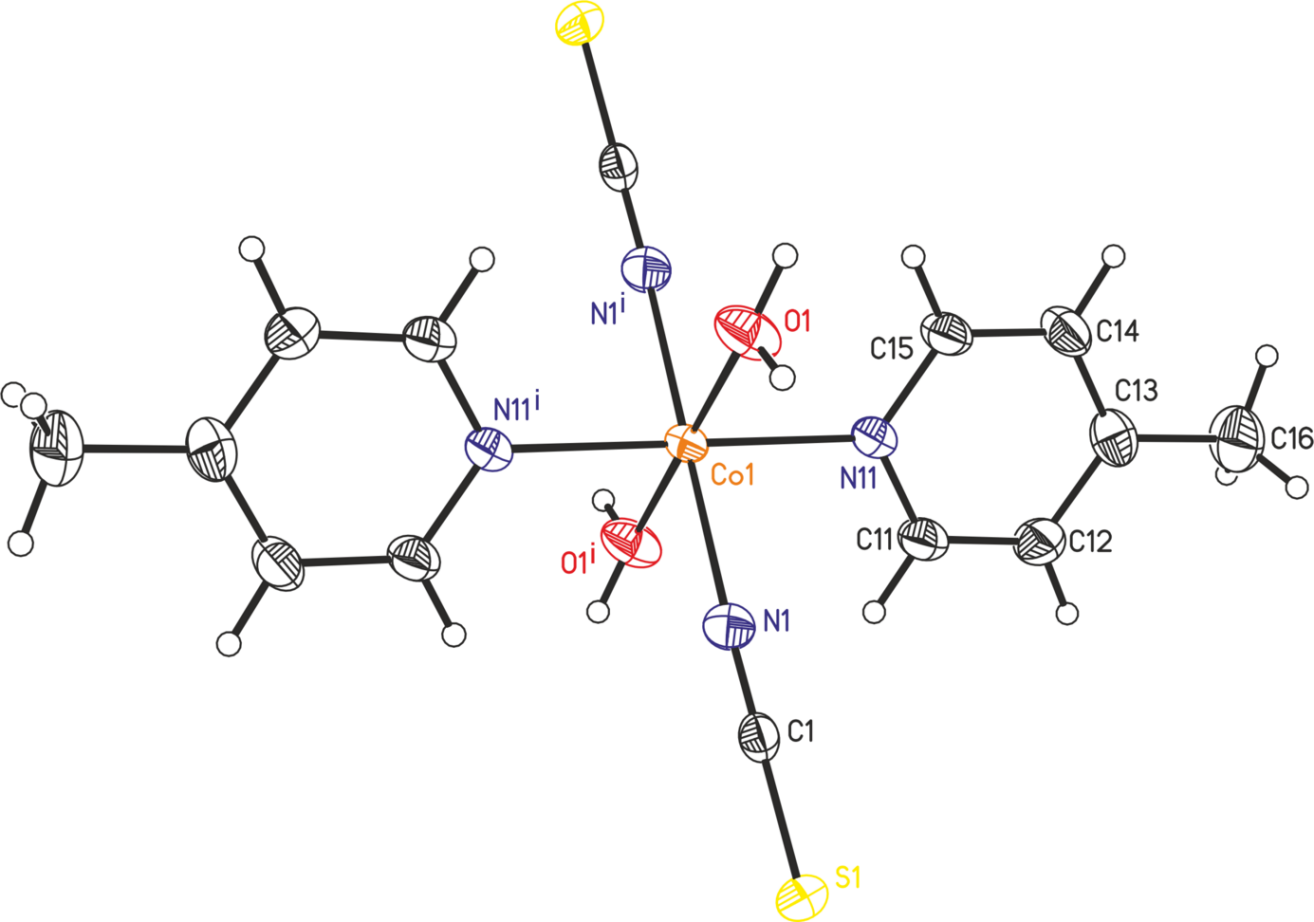
The mol­ecular structure of the title compound with displacement ellipsoids drawn at the 50% probability level. Symmetry code: (i) −*x* + 1, −*y*, −*z*.

The asymmetric unit of the title compound, Co(NCS)_2_(C_6_H_7_N)_2_(H_2_O)_2_ (C_6_H_7_N = 4-methyl­pyridine) is built up of one cobalt cation located on a crystallographic inversion centre as well as one thio­cyanate anion, one 4-methyl­pyridine ligand and one water mol­ecule that occupy general positions (Fig. 1 ▸). The Co cation is therefore sixfold coordinated in a *trans*-CoN_4_O_2_ geometry by two terminal N-bonded thio­cyanate anions, two 4-methyl­pyridine ligands and two water mol­ecules (Fig. 1 ▸). The bonding angles around the Co centers deviate from the ideal values, which means that the octa­hedra are slightly distorted (Table 1 ▸).

It is noted that the title compound is isotypic to Fe(NCS)_2_(4-methyl­pyridine)_2_(H_2_O)_2_ (Cambridge Structural Database refcode VUCCEE; Neumann *et al.*, 2020*a* ▸) and Mn(NCS)_2_(4-methyl­pyridine)_2_(H_2_O)_2_ (VUJYAD; Neumann *et al.*, 2020*b* ▸) reported in the literature. That the chloro-methyl-exchange is also valid for these compounds is shown by the fact that the title compound is also isotypic to Ni(NCS)_2_(4-chloro­pyridine)_2_(H_2_O)_2_ (GIQQEF; Jochim *et al.*, 2018 ▸) and Co(NCS)_2_(4-chloro­pyridine)_2_(H_2_O)_2_ (UHUVEB; Böhme *et al.*, 2020 ▸).

## 3. Supra­molecular features

**Figure 2:**
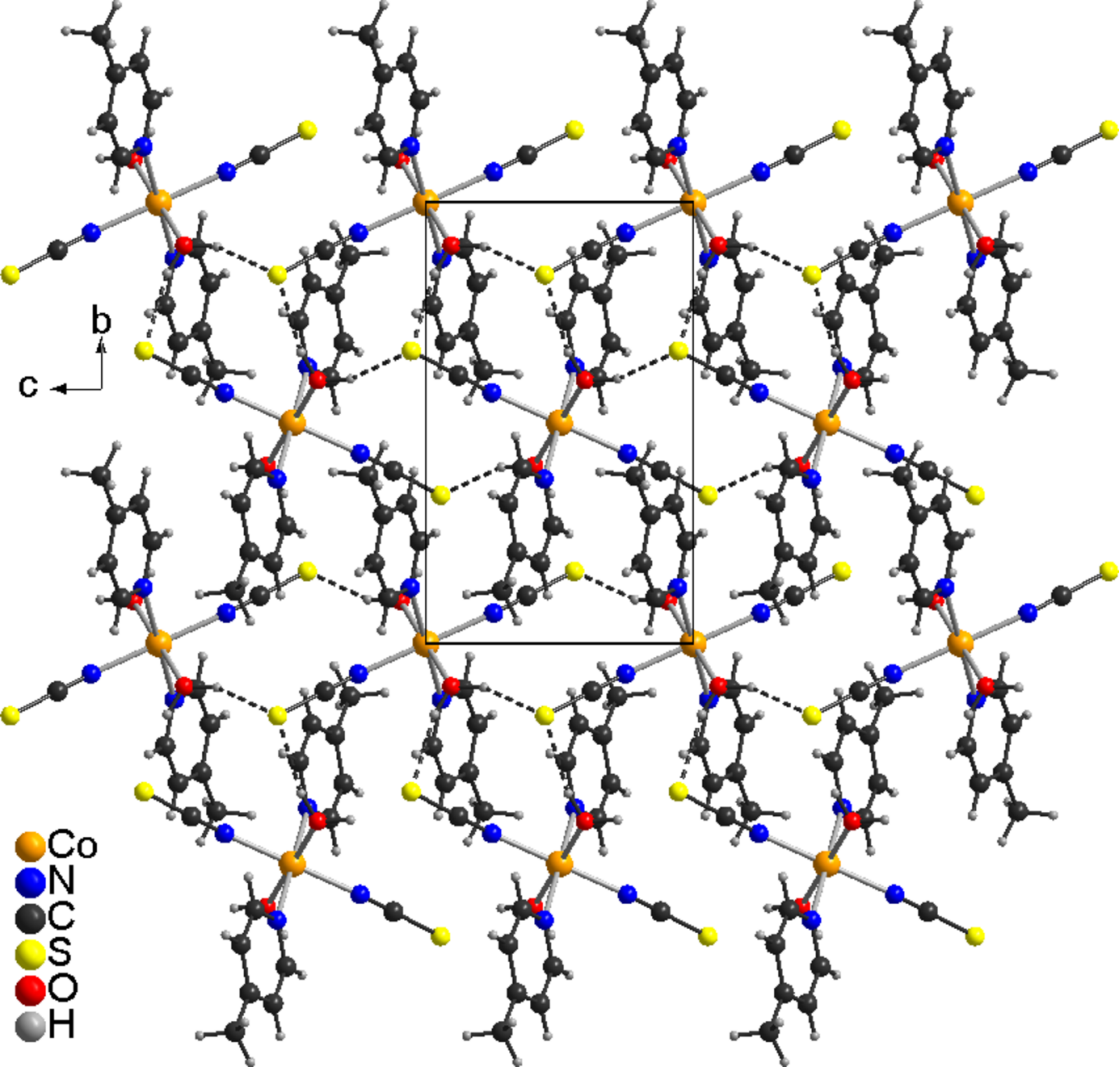
Crystal structure of the title compound in a view along the *a*-axis direction. O—H⋯S hydrogen bonds are shown as dashed lines.

**Figure 3:**
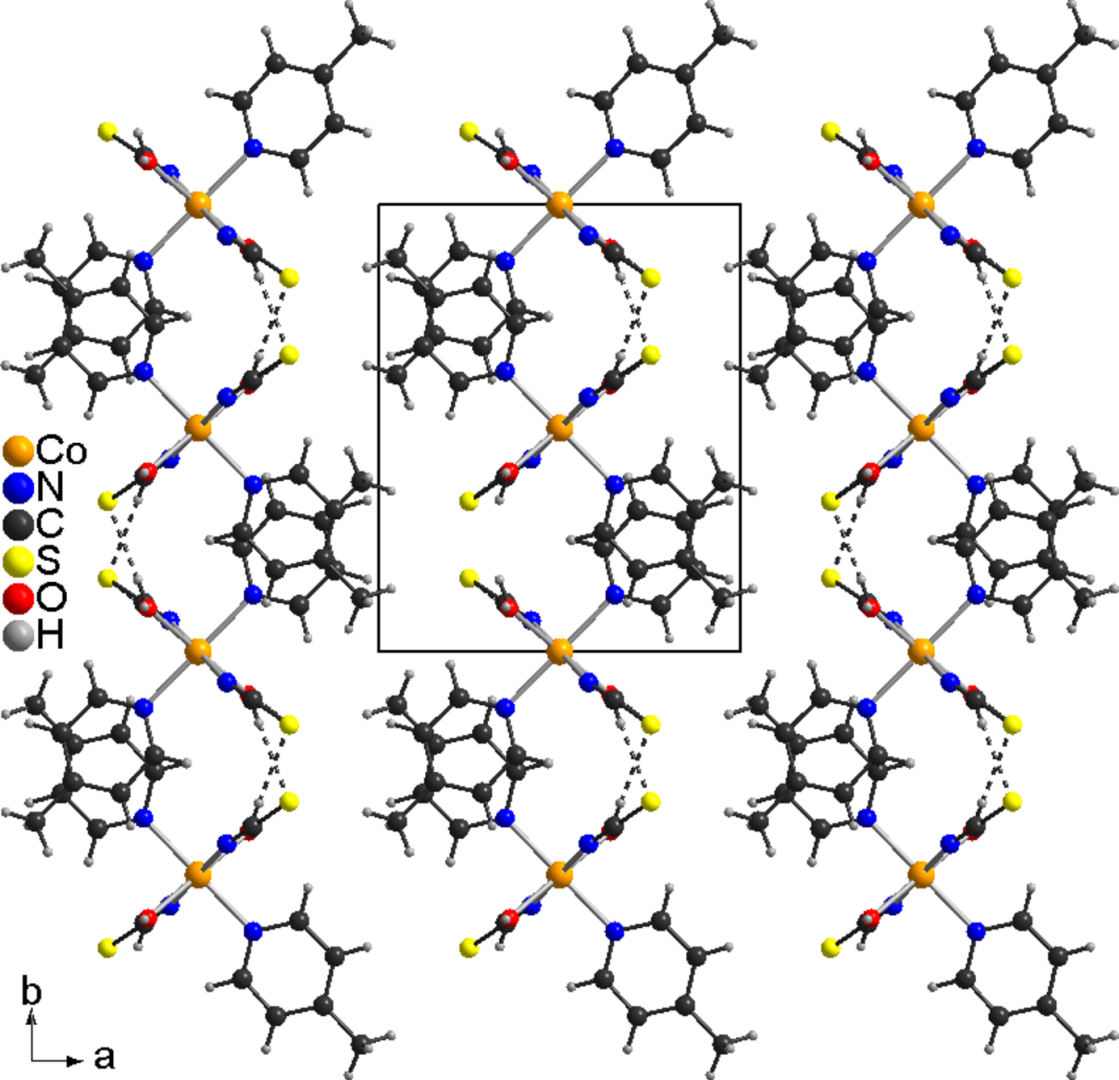
Crystal structure of the title compound in a view along the *c*-axis direction. O—H⋯S hydrogen bonding is shown as dashed lines.

In the extended structure of the title compound, the complexes are linked by O—H⋯S hydrogen bonds into layers that lie parallel to the *bc*-plane (Fig. 2 ▸). The O—H⋯S bond angles are close to linear, which indicates that these are relatively strong inter­actions (Table 2 ▸). Between the layers, no pronounced directional inter­actions are observed (Fig. 3 ▸).

## 4. Thermal properties

**Figure 4:**
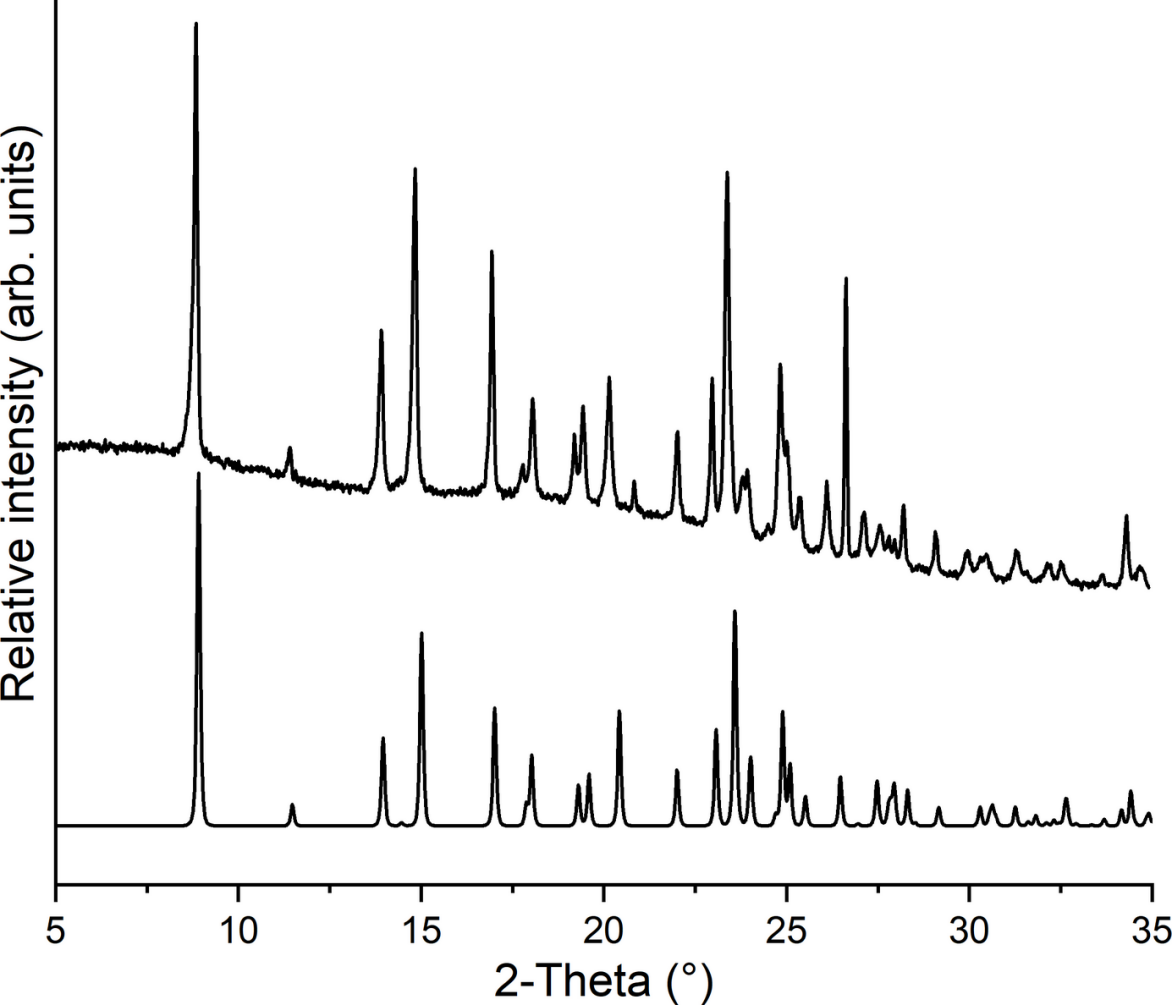
Experimental (top) and calculated (bottom) PXRD patterns for the title compound.

Comparison of the experimental powder pattern of the residue obtained in the synthesis with that calculated for the title compound shows that a pure crystalline phase has been obtained (Fig. 4 ▸).

**Figure 5:**
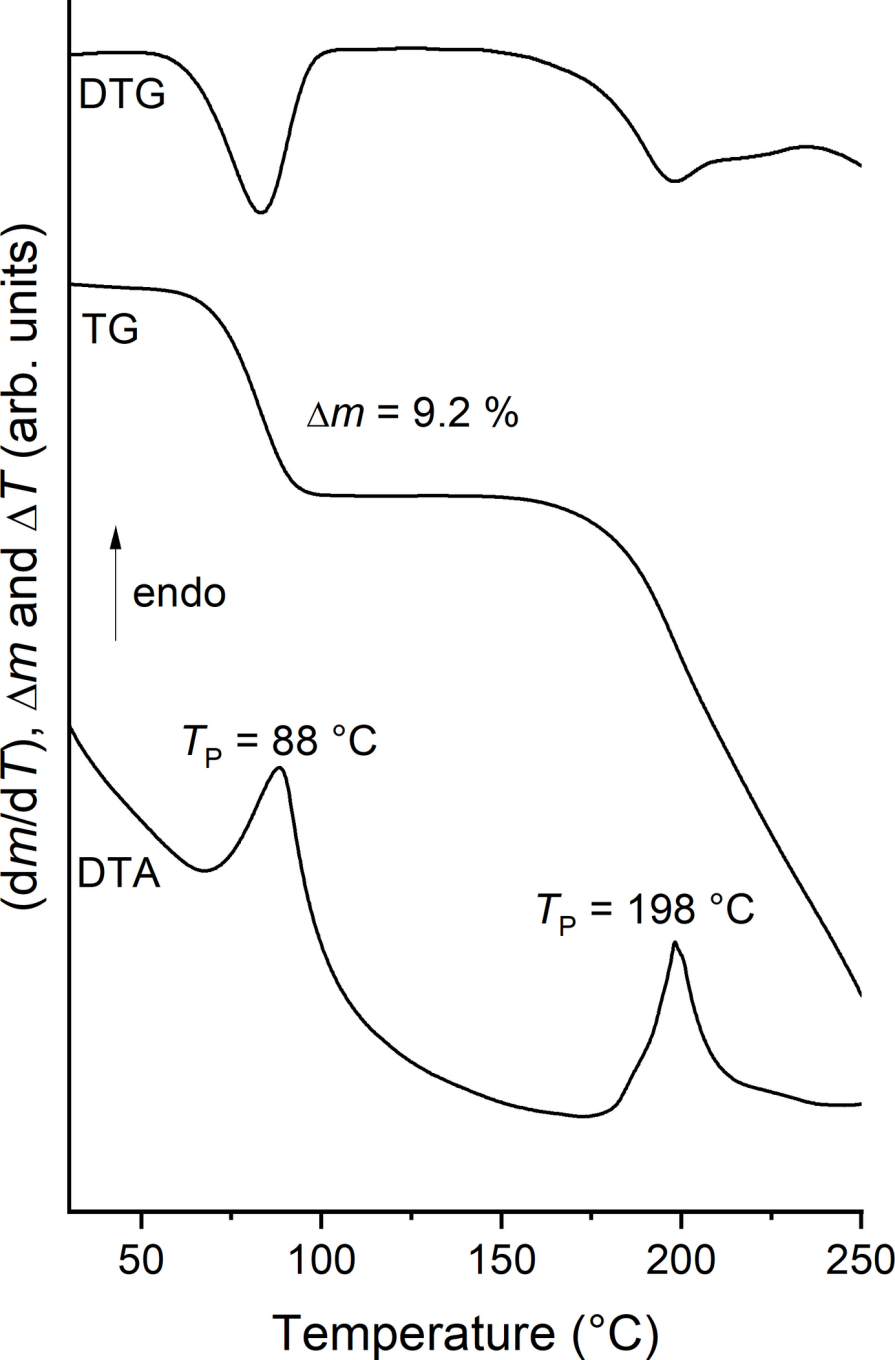
DTG, TG and DTA curves for the title compound. The mass loss is given in % and the peak temperature in °C.

**Figure 6:**
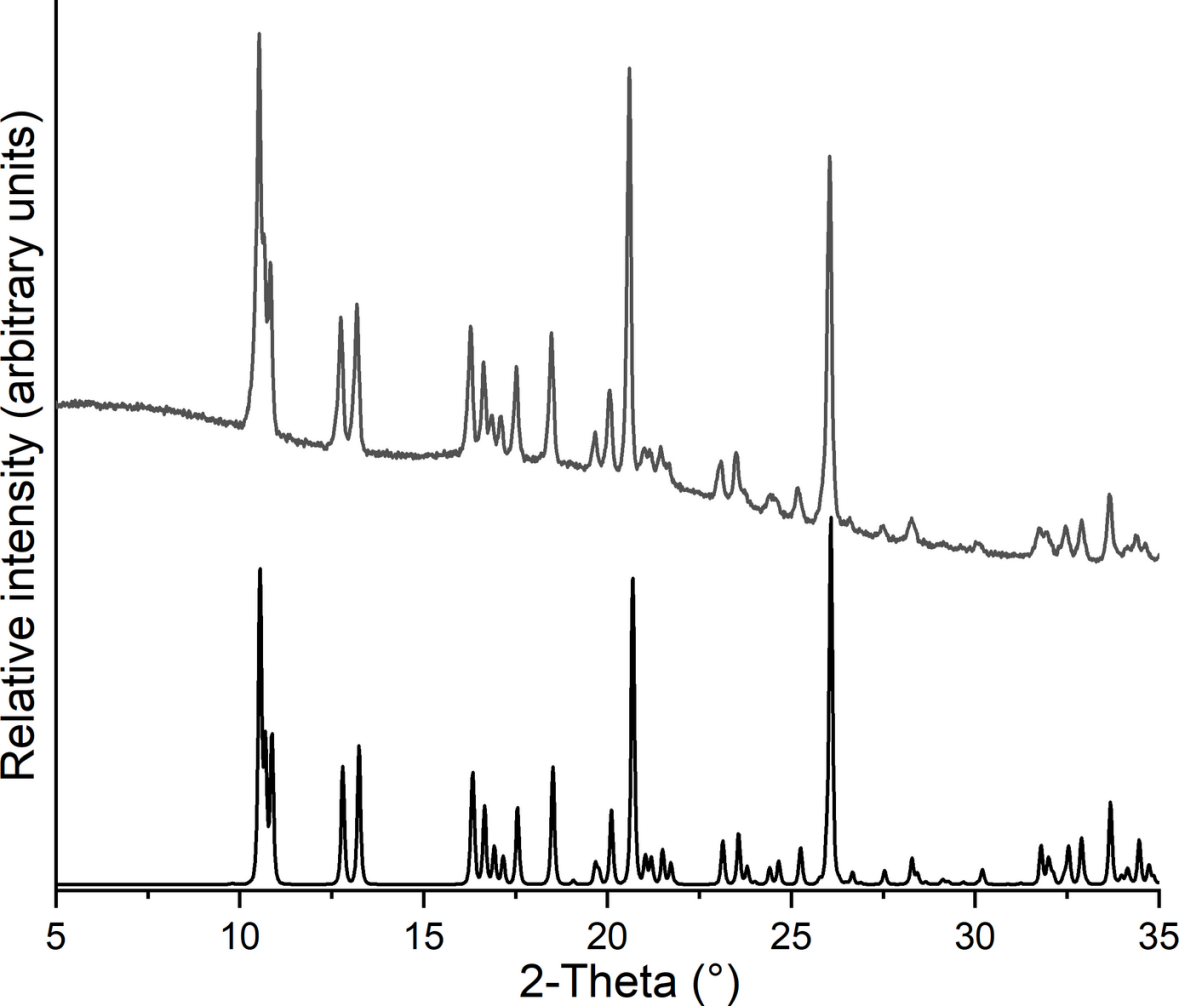
Experimental PXRD pattern of the residue obtained after the first mass loss in a TG measurement of the title compound (top) and the powder pattern for Co(NCS)_2_(4-methyl­pyridine)_2_ calculated from single-crystal data (bottom, Näther & Boeckmann, 2025 ▸).

The thermal properties of the title compound were investigated by differential thermoanalysis coupled to thermogravimetry (DTA–TG). Upon heating, one mass loss is observed in the TG curve accompanied with an endothermic event in the DTA curve at 88°C (Fig. 5 ▸). On further heating, the sample mass decreases continuously with a further poorly resolved mas-losss step, which is apparent in the DTG curve and which is accompanied with a second endothermic event in the DTA curve. The experimental mass loss in the first step of 9.2% is in good agreement with that calculated for the removal of the two water mol­ecules (9.1%). Comparison of the experimental powder pattern of the residue obtained after the first mass loss shows that the monoclinic form with linear chains of Co(NCS)_2_(4-methyl­pyridine)_2_ (Näther & Boeckmann, 2025 ▸) has formed, which can also be obtained from solution (Fig. 6 ▸). This is in contrast to the observations made for the corresponding 4-chloro­pyridine compound (see *Chemical context*) and indicates that with 4-methyl­pyridine only one isomer might be accessible.

## 5. Database survey

According to a search in the CSD (version 5.43, last update December 2024; Groom *et al.*, 2016 ▸) using CONQUEST (Bruno *et al.*, 2002 ▸), some compounds containing Co(NCS)_2_ and 4-methyl­pyridine have already been reported in the literature. These include a discrete complex with the composition CoNCS(4-methyl­pyridine)_3_ with *p*-xylene solvate mol­ecules (QQQGKJ; Solaculu *et al.*, 1974 ▸), but because no atomic coordinates are given and no charge balance is achieved, the existence of this compound is questionable. There is also a compound with the composition Co(NCS)_2_(4-methyl­pyridine)_2_-bis­(*p*-toluidine)_2_, which should consists of chains, but even for this structure no atomic coordinates are reported (Refcode: CECDAP; Micu-Semeniuc *et al.*, 1983 ▸).

All of the remaining hits describe discrete complexes with the composition Co(NCS)_2_(4-methyl­pyridine)_4_, which forms clathrates with 4-methyl­pyridine (XIHHEB; Harris *et al.*, 2001 ▸ and XIHHEB01; Harris *et al.*, 2003 ▸), nitro­benzene (ZZZUXU; Belitskus *et al.*, 1963 ▸), *p*-toluidine (CECCOC; Micu-Semeniuc *et al.*, 1983 ▸), nitro­ethane (ZZZUXY; Belitskus *et al.*, 1963 ▸) and benzene (ZZZUYI; Belitskus *et al.*, 1963 ▸). With one exception (XIHHEB), no atomic coordinates are presented for any of these compounds. The crystal structure of the complex Co(NCS)_2_(4-methyl­pyridine)_4_ is also found, but the unit-cell parameters are almost identical to that of several of the clathrates mentioned above, indicating that a potential solvent was not located (VERNUC; Harris *et al.*, 2003 ▸).

## 6. Synthesis and crystallization

Co(NCS)_2_ (350.2 mg, 2.06 mmol, Alfa Aesar) and 4-methyl­pyridine (100 µl, 1.03 mmol, Fluka) were stirred together in 3 ml of water at room temperature in a snap cap vial for 3 d. Single crystals were prepared by the same method in the absence of stirring.

Powder X-ray diffraction measurements were performed using a Stoe STADI P transmission powder diffractometer with Cu *K*α_1_ radiation (λ = 1.540598 Å), a Johann-type Ge(111) monochromator and a MYTHEN 1K detector from Dectris. Thermogravimetry and differential thermoanalysis (TG–DTA) measurements were performed in a dynamic nitro­gen atmosphere in Al_2_O_3_ crucibles with a heating rate of 4°C min^−1^ using a STA-PT 1000 thermobalance from Linseis. The TG–DTA instrument was calibrated using standard reference materials.

## 7. Refinement

Crystal data, data collection and structure refinement details are summarized in Table 3 ▸. The C-bound hydrogen atoms were positioned with idealized geometry (methyl H atoms allowed to rotate and not to tip) and were refined with *U*_iso_(H) = 1.2*U*_eq_(C) (1.5 for methyl H atoms) using a riding model. The O-bound H atoms were located in a difference map and their positions and *U*_iso_ values were freely refined. The crystal chosen for data collection was twinned and therefore a twin refinement using data in HKLF-5 format was used leading to a BASF factor of 0.173 (3) for the minor twin component.

## Supplementary Material

Crystal structure: contains datablock(s) I. DOI: 10.1107/S2056989025003469/hb8129sup1.cif

Structure factors: contains datablock(s) I. DOI: 10.1107/S2056989025003469/hb8129Isup2.hkl

CCDC reference: 2444751

Additional supporting information:  crystallographic information; 3D view; checkCIF report

## Figures and Tables

**Table 1:** Selected geometric parameters (Å, °)

Co1—N1	2.090 (3)	Co1—N11	2.162 (2)
Co1—O1	2.105 (2)		
			
N1^i^—Co1—O1	86.81 (10)	O1—Co1—N11^i^	88.16 (9)
N1—Co1—O1	93.19 (10)	O1—Co1—N11	91.84 (9)
N1^i^—Co1—N11	90.12 (10)	C1—N1—Co1	163.9 (3)
N1—Co1—N11	89.88 (10)		

Symmetry code: (i) 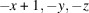.

**Table 2:** Hydrogen-bond geometry (Å, °)

*D*—H⋯*A*	*D*—H	H⋯*A*	*D*⋯*A*	*D*—H⋯*A*
O1—H1*A*⋯S1^ii^	0.96 (4)	2.35 (4)	3.234 (3)	153 (3)
O1—H1*B*⋯S1^iii^	0.93 (5)	2.35 (5)	3.252 (2)	164 (5)
C12—H12⋯S1^iv^	0.95	2.91	3.833 (3)	164

Symmetry codes: (ii) 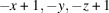; (iii) 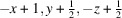; (iv) 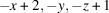.

**Table 3:** Experimental details

Crystal data	
Chemical formula	[Co(NCS)_2_(C_6_H_7_N)_2_(H_2_O)_2_]
*M*_r_	397.37
Crystal system, space group	Monoclinic, *P*2_1_/*c*
Temperature (K)	200
*a*, *b*, *c* (Å)	10.1901 (6), 12.2379 (10), 7.6165 (9)
β (°)	103.379 (10)
*V* (Å^3^)	924.04 (15)
*Z*	2
Radiation type	Mo *K*α
μ (mm^−1^)	1.17
Crystal size (mm)	0.30 × 0.22 × 0.21

Data collection	
Diffractometer	Stoe IPDS2
Absorption correction	Numerical (*X-SHAPE* and *X-RED32*; Stoe, 2008 ▸)
*T*_min_, *T*_max_	0.793, 0.813
No. of measured, independent and observed [*I* > 2σ(*I*)] reflections	2179, 2179, 1822
*R*_int_	?
(sin θ/λ)_max_ (Å^−1^)	0.662

Refinement	
*R*[*F*^2^ > 2σ(*F*^2^)], *wR*(*F*^2^), *S*	0.047, 0.143, 1.04
No. of reflections	2179
No. of parameters	117
H-atom treatment	H atoms treated by a mixture of independent and constrained refinement
Δρ_max_, Δρ_min_ (e Å^−3^)	1.23, −0.56

Computer programs: *X-AREA* (Stoe, 2008 ▸), *SHELXT* (Sheldrick, 2015*a* ▸), *SHELXL* (Sheldrick, 2015*b* ▸), *DIAMOND* (Brandenburg, 1999 ▸), *XP* in *SHELXTL-PC* (Sheldrick, 2008 ▸) and *publCIF* (Westrip, 2010 ▸).

## supplementary crystallographic information

### Diaquabis(4-methylpyridine-κN)bis(thiocyanato-κN)cobalt(II) Crystal data

**Table:**

[Co(NCS)_2_(C_6_H_7_N)_2_(H_2_O)_2_]	*F*(000) = 410
*M**_r_* = 397.37	*D*_x_ = 1.428 Mg m^−^^3^
Monoclinic, *P*2_1_/*c*	Mo *K*α radiation, λ = 0.71073 Å
*a* = 10.1901 (6) Å	Cell parameters from 5888 reflections
*b* = 12.2379 (10) Å	θ = 2.6–28.1°
*c* = 7.6165 (9) Å	µ = 1.17 mm^−^^1^
β = 103.379 (10)°	*T* = 200 K
*V* = 924.04 (15) Å^3^	Block, light blue
*Z* = 2	0.30 × 0.22 × 0.21 mm

### Diaquabis(4-methylpyridine-κN)bis(thiocyanato-κN)cobalt(II) Data collection

**Table:**

Stoe IPDS-2 diffractometer	1822 reflections with *I* > 2σ(*I*)
ω scans	θ_max_ = 28.1°, θ_min_ = 2.6°
Absorption correction: numerical (X-Shape and X-Red32; Stoe, 2008)	*h* = −13→13
*T*_min_ = 0.793, *T*_max_ = 0.813	*k* = −16→16
2179 measured reflections	*l* = 0→9
2179 independent reflections	

### Diaquabis(4-methylpyridine-κN)bis(thiocyanato-κN)cobalt(II) Refinement

**Table:**

Refinement on *F*^2^	Hydrogen site location: mixed
Least-squares matrix: full	H atoms treated by a mixture of independent and constrained refinement
*R*[*F*^2^ > 2σ(*F*^2^)] = 0.047	*w* = 1/[σ^2^(*F*_o_^2^) + (0.1088*P*)^2^] where *P* = (*F*_o_^2^ + 2*F*_c_^2^)/3
*wR*(*F*^2^) = 0.143	(Δ/σ)_max_ < 0.001
*S* = 1.04	Δρ_max_ = 1.23 e Å^−^^3^
2179 reflections	Δρ_min_ = −0.56 e Å^−^^3^
117 parameters	Extinction correction: *SHELXL2016/6* (Sheldrick 2015b), Fc^*^=kFc[1+0.001xFc^2^λ^3^/sin(2θ)]^-1/4^
0 restraints	Extinction coefficient: 0.044 (9)
Primary atom site location: dual	

### Diaquabis(4-methylpyridine-κN)bis(thiocyanato-κN)cobalt(II) Special details

**Table:**

Geometry. All esds (except the esd in the dihedral angle between two l.s. planes) are estimated using the full covariance matrix. The cell esds are taken into account individually in the estimation of esds in distances, angles and torsion angles; correlations between esds in cell parameters are only used when they are defined by crystal symmetry. An approximate (isotropic) treatment of cell esds is used for estimating esds involving l.s. planes.
Refinement. Refined as a 2-component twin.

### Diaquabis(4-methylpyridine-κN)bis(thiocyanato-κN)cobalt(II) Fractional atomic coordinates and isotropic or equivalent isotropic displacement parameters (Å^2^)

**Table:**

	*x*	*y*	*z*	*U*_iso_*/*U*_eq_	
Co1	0.500000	0.000000	0.000000	0.0221 (2)	
N1	0.5788 (3)	−0.0691 (2)	0.2534 (4)	0.0319 (6)	
C1	0.6496 (3)	−0.1080 (2)	0.3783 (4)	0.0227 (6)	
S1	0.75122 (7)	−0.16385 (6)	0.55520 (11)	0.0295 (2)	
O1	0.3604 (2)	0.09689 (18)	0.0952 (4)	0.0357 (5)	
H1A	0.351 (4)	0.100 (3)	0.218 (6)	0.032 (10)*	
H1B	0.333 (5)	0.161 (4)	0.032 (7)	0.061 (14)*	
N11	0.6515 (2)	0.12690 (18)	0.0581 (4)	0.0262 (5)	
C11	0.7790 (3)	0.1036 (2)	0.1405 (5)	0.0327 (7)	
H11	0.803034	0.029241	0.164939	0.039*	
C12	0.8776 (3)	0.1823 (3)	0.1920 (5)	0.0352 (7)	
H12	0.966985	0.161295	0.249329	0.042*	
C13	0.8462 (3)	0.2924 (2)	0.1600 (5)	0.0326 (7)	
C14	0.7142 (3)	0.3165 (2)	0.0721 (5)	0.0326 (7)	
H14	0.687692	0.390105	0.044934	0.039*	
C15	0.6213 (3)	0.2326 (2)	0.0241 (5)	0.0294 (6)	
H15	0.531593	0.251015	−0.036086	0.035*	
C16	0.9504 (4)	0.3801 (3)	0.2155 (7)	0.0495 (10)	
H16A	0.916053	0.449122	0.157288	0.074*	
H16B	1.032996	0.359451	0.178640	0.074*	
H16C	0.970268	0.389082	0.346878	0.074*	

### Diaquabis(4-methylpyridine-κN)bis(thiocyanato-κN)cobalt(II) Atomic displacement parameters (Å^2^)

**Table:**

	*U*^11^	*U*^22^	*U*^33^	*U*^12^	*U*^13^	*U*^23^
Co1	0.0262 (3)	0.0165 (3)	0.0219 (3)	0.00175 (17)	0.0019 (2)	0.00247 (18)
N1	0.0395 (13)	0.0261 (11)	0.0262 (13)	−0.0018 (10)	−0.0002 (11)	0.0060 (10)
C1	0.0284 (12)	0.0190 (11)	0.0211 (13)	−0.0054 (9)	0.0068 (10)	−0.0017 (10)
S1	0.0286 (4)	0.0301 (4)	0.0255 (4)	−0.0001 (3)	−0.0025 (3)	0.0029 (3)
O1	0.0472 (13)	0.0261 (10)	0.0374 (14)	0.0112 (9)	0.0171 (11)	0.0031 (9)
N11	0.0259 (11)	0.0191 (10)	0.0314 (13)	0.0022 (8)	0.0024 (10)	0.0020 (9)
C11	0.0273 (14)	0.0238 (13)	0.0430 (19)	0.0030 (11)	−0.0001 (13)	0.0049 (12)
C12	0.0247 (13)	0.0330 (15)	0.0435 (19)	0.0000 (12)	−0.0011 (12)	0.0037 (14)
C13	0.0332 (15)	0.0275 (14)	0.0400 (18)	−0.0062 (12)	0.0140 (13)	−0.0024 (12)
C14	0.0343 (14)	0.0200 (12)	0.0437 (19)	−0.0007 (11)	0.0094 (13)	0.0022 (12)
C15	0.0252 (12)	0.0221 (12)	0.0399 (17)	0.0035 (10)	0.0055 (12)	0.0049 (12)
C16	0.0386 (18)	0.0418 (19)	0.065 (3)	−0.0149 (15)	0.0063 (17)	−0.0028 (18)

### Diaquabis(4-methylpyridine-κN)bis(thiocyanato-κN)cobalt(II) Geometric parameters (Å, º)

**Table:**

Co1—N1	2.090 (3)	C11—H11	0.9500
Co1—N1^i^	2.090 (3)	C11—C12	1.381 (4)
Co1—O1^i^	2.105 (2)	C12—H12	0.9500
Co1—O1	2.105 (2)	C12—C13	1.393 (4)
Co1—N11^i^	2.162 (2)	C13—C14	1.387 (4)
Co1—N11	2.162 (2)	C13—C16	1.501 (4)
N1—C1	1.155 (4)	C14—H14	0.9500
C1—S1	1.646 (3)	C14—C15	1.387 (4)
O1—H1A	0.96 (4)	C15—H15	0.9500
O1—H1B	0.93 (5)	C16—H16A	0.9800
N11—C11	1.337 (4)	C16—H16B	0.9800
N11—C15	1.341 (3)	C16—H16C	0.9800
			
N1—Co1—N1^i^	180.0	C15—N11—Co1	122.18 (19)
N1^i^—Co1—O1	86.81 (10)	N11—C11—H11	118.3
N1^i^—Co1—O1^i^	93.19 (10)	N11—C11—C12	123.3 (3)
N1—Co1—O1	93.19 (10)	C12—C11—H11	118.3
N1—Co1—O1^i^	86.81 (10)	C11—C12—H12	119.9
N1^i^—Co1—N11^i^	89.88 (10)	C11—C12—C13	120.1 (3)
N1^i^—Co1—N11	90.12 (10)	C13—C12—H12	119.9
N1—Co1—N11	89.88 (10)	C12—C13—C16	121.6 (3)
N1—Co1—N11^i^	90.12 (10)	C14—C13—C12	116.6 (3)
O1—Co1—O1^i^	180.0	C14—C13—C16	121.9 (3)
O1—Co1—N11^i^	88.16 (9)	C13—C14—H14	120.1
O1^i^—Co1—N11	88.16 (9)	C15—C14—C13	119.8 (3)
O1—Co1—N11	91.84 (9)	C15—C14—H14	120.1
O1^i^—Co1—N11^i^	91.84 (9)	N11—C15—C14	123.4 (3)
N11^i^—Co1—N11	180.00 (9)	N11—C15—H15	118.3
C1—N1—Co1	163.9 (3)	C14—C15—H15	118.3
N1—C1—S1	179.5 (3)	C13—C16—H16A	109.5
Co1—O1—H1A	126 (2)	C13—C16—H16B	109.5
Co1—O1—H1B	116 (3)	C13—C16—H16C	109.5
H1A—O1—H1B	113 (4)	H16A—C16—H16B	109.5
C11—N11—Co1	120.91 (18)	H16A—C16—H16C	109.5
C11—N11—C15	116.8 (2)	H16B—C16—H16C	109.5
			
Co1—N11—C11—C12	−175.4 (3)	C11—C12—C13—C16	179.5 (4)
Co1—N11—C15—C14	175.0 (3)	C12—C13—C14—C15	1.1 (5)
N11—C11—C12—C13	0.5 (6)	C13—C14—C15—N11	0.1 (6)
C11—N11—C15—C14	−0.9 (5)	C15—N11—C11—C12	0.6 (5)
C11—C12—C13—C14	−1.4 (5)	C16—C13—C14—C15	−179.7 (4)

Symmetry code: (i) −*x*+1, −*y*, −*z*.

### Diaquabis(4-methylpyridine-κN)bis(thiocyanato-κN)cobalt(II) Hydrogen-bond geometry (Å, º)

**Table:**

*D*—H···*A*	*D*—H	H···*A*	*D*···*A*	*D*—H···*A*
O1—H1*A*···S1^ii^	0.96 (4)	2.35 (4)	3.234 (3)	153 (3)
O1—H1*B*···S1^iii^	0.93 (5)	2.35 (5)	3.252 (2)	164 (5)
C12—H12···S1^iv^	0.95	2.91	3.833 (3)	164

Symmetry codes: (ii) −*x*+1, −*y*, −*z*+1; (iii) −*x*+1, *y*+1/2, −*z*+1/2; (iv) −*x*+2, −*y*, −*z*+1.
